# PKM2 promotes metastasis by recruiting myeloid-derived suppressor cells and indicates poor prognosis for hepatocellular carcinoma

**DOI:** 10.18632/oncotarget.2749

**Published:** 2014-12-02

**Authors:** Wei-Ren Liu, Meng-Xin Tian, Liu-Xiao Yang, Yu-Li Lin, Lei Jin, Zhen-Bin Ding, Ying-Hao Shen, Yuan-Fei Peng, Dong-Mei Gao, Jian Zhou, Shuang-Jian Qiu, Zhi Dai, Rui He, Jia Fan, Ying-Hong Shi

**Affiliations:** ^1^ Department of Liver Surgery, Liver Cancer Institute, Zhongshan Hospital, Fudan University; Key Laboratory of Carcinogenesis and Cancer Invasion of Ministry of Education, Shanghai, People's Republic of China; ^2^ Department of Immunology, School of Basic Medical Sciences, Shanghai Medical College, Fudan University, Shanghai, People's Republic of China; ^3^ Institutes of Biomedical Sciences, Fudan University, Shanghai, People's Republic of China

**Keywords:** PKM2, Myeloid-derived Suppressor Cells, Prognosis, Hepatocellular Carcinoma, Metastasis

## Abstract

Pyruvate kinase M2 (PKM2) is a member of the pyruvate kinase family. Recent work has defined the “non-metabolic” functions of PKM2. However, the role of PKM2 in HCC remains unclear. To investigate the role of PKM2 in tumor growth, invasion and the prognosis of hepatocellular carcinoma (HCC), PKM2 expression was measured in HCC cell lines and tissues using qRT-PCR, western blot, and immunofluorescence assays. In *in vitro* experiments, PKM2 was knocked down using a short hairpin RNA lentivirus vector, and tumor cell behavior and the downstream signaling pathways and chemokine were analyzed. For the analysis of *in vivo* tumor growth, intratumoral and peritumoral lymphocyte infiltration were examined in nude mice. The prognostic value of PKM2 was analyzed by immunohistochemistry in two cohorts including 721 HCC patients. Together, our data obtained from cell lines, tumorigenicity studies, and primary HCC samples illustrate an oncogenic role for PKM2 in tumors. Moreover, PKM2 may serve as a novel prognostic indicator for HCC patients after curative resection, targeted therapy aimed at PKM2 may represent an effective treatment approach for HCC.

## INTRODUCTION

Hepatocellular carcinoma (HCC) is the second most common cause of cancer-related death worldwide, and the burden of this disease is especially high in developing countries [1]. Although significant HCC research has focused on oncogenes and tumor suppressor genes, the importance of cancer cell metabolism has been recently highlighted [2]. Cancer cell metabolism is known as the Warburg effect [3, 4], whereby cancer cells shift their glucose metabolism largely to glycolysis via a process called “aerobic glycolysis.”

A key glycolytic enzyme that is consistently altered during tumorigenesis is pyruvate kinase (PK). PK converts phosphoenolpyruvate (PEP) and ADP to pyruvate and ATP and regulates glucose carbon flux in the cell [5]. PK is composed of 4 isoenzymes: L, R, M1, and M2. Types L and R are expressed in the liver and erythrocytes; type M1 is present in most normal tissue; and type M2 is characteristic of all proliferating cells, especially tumor cells [6]. PKM1 and PKM2 represent different splicing products of the same mRNA transcript, but they differ in terms of 21 amino acids [7].

The non-metabolic function of PKM2 is emerging in cancer. For example, PKM2 knockdown resulted in a reduced ability to form tumors in a nude mouse xenograft model [8]. PKM2 was also shown to be critical for rapid growth in cancer cells by binding directly and selectively to tyrosine-phosphorylated peptides [9]. PKM2 interacted with SOCS3, which led to reduced production of ATP and impaired DC-based immunotherapy against tumors [10]. PKM2 reprogrammed glucose metabolism in cancer cells by participating in a positive feedback loop that promoted HIF-1 transactivation [11]. Furthermore, the disruption of PKM2 was shown to suppress oncogenic mTOR-mediated tumorigenesis, and the PKM2 signaling pathway was identified as a potential target for the treatment of cancer [12]. Several siRNAs targeting PKM2 had led to decreased viability and increased apoptosis in multiple cancer cell lines, and the *in vivo* delivery of siPKM2 led to substantial tumor regression of established xenografts [5]. However, few published reports have described the role of PKM2 in HCC. Although PKM2 mRNA expression was closely related to proliferative activity in HCC [13], the role of PKM2 in HCC and the mechanism responsible for the oncogenic role of PKM2 remain unknown.

In the present study, we investigated the expression of PKM2 in a series of metastatic HCC cell lines, and our results provide evidence for the oncogenic role of PKM2 in HCC *in vitro* and *in vivo*. We also found that PKM2 expression was associated with the production of chemotactic factors that induced myeloid cell infiltration in tumor xenografts. Finally, using tissue microarrays (TMAs) with HCC samples, our findings revealed the prognostic value of PKM2.

## RESULTS

### PKM2 is overexpressed in human cancers

**Figure 1:**
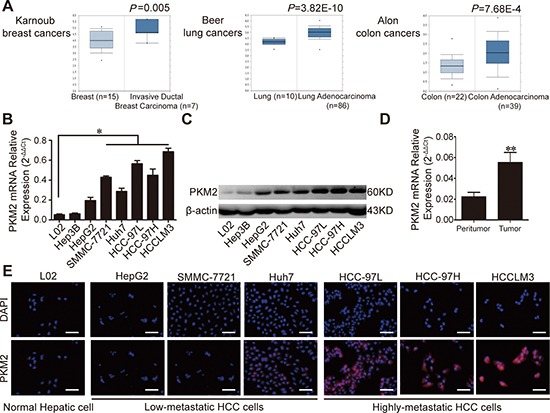
Expression of PKM2 in human cancers and HCC tissues/cell lines **(A)** Microarray data analyses of PKM2 expression in human cancers are shown. PKM2 mRNA levels in human normal/cancer tissues (breast, lung, and colon) are plotted. The Student t test was conducted using the Oncomine software. The boxes represent the 25th through 75th percentiles. The horizontal lines represent the medians. The whiskers represent the 10th and 90th percentiles, and the asterisks represent the end of the ranges. **(B)** Quantitative RT-PCR confirmed different PKM2 mRNA expression in HCC cell lines. Data shown as mean (± SD) from three independent experiments. **(C)** Expression of PKM2 in normal human liver epithelial cells (L02) and 7 HCC cell lines was examined using Western blotting; β-actin was used as a loading control. **(D)** Quantitative RT-PCR analysis of PKM2 levels in HCC tissues and corresponding peritumoral tissue. β-actin was used as a control. Error bars indicate standard deviation (SD) (*n* = 48). **(E)** Fluorescence microscopic analysis for PKM2 expression. *P < 0.05. **P < 0.01.

We first compared the expression levels of PKM2 using the Oncomine database, which includes publicly available cDNA microarray data from cancer samples. Analysis of representative data sets indicated that the PKM2 mRNA levels were higher in liver cancer tissues compared to peritumoral tissues [14] (data not shown). We also analyzed the gene expression profiles of other cancers and found that the PKM2 mRNA levels were also elevated in breast cancer [15], lung cancer [16], colon cancer [17] (Fig. 1A), gastric cancer [18], and bladder cancer [19] (data not shown). Thus, increased PKM2 expression is associated with multiple human cancers.

We next examined PKM2 expression in a series of HCC cell lines with increasing metastatic potential (L02, Hep3B, HepG2, SMMC-7721, Huh7, HCC-97L, HCC-97H, and HCCLM3). The trend of increased PKM2 mRNA and protein expression in cancer cells was consistent with the metastatic potential (Fig. 1B, 1C). In line with the Oncomine data, the average PKM2 mRNA expression level was significantly higher in tumor tissues compared with peritumoral tissues (Fig. 1D). In particular, MHCC97L, MHCC97H, and HCCLM3 cells displayed strong immunostaining, especially in the cytoplasm, in contrast to Huh7, SMMC-7721, HepG2, and L02 cells, which displayed low levels of fluorescence (Fig. 1E).

### PKM2 knockdown inhibits the tumor progression of Hcc *in vitro*

**Figure 2:**
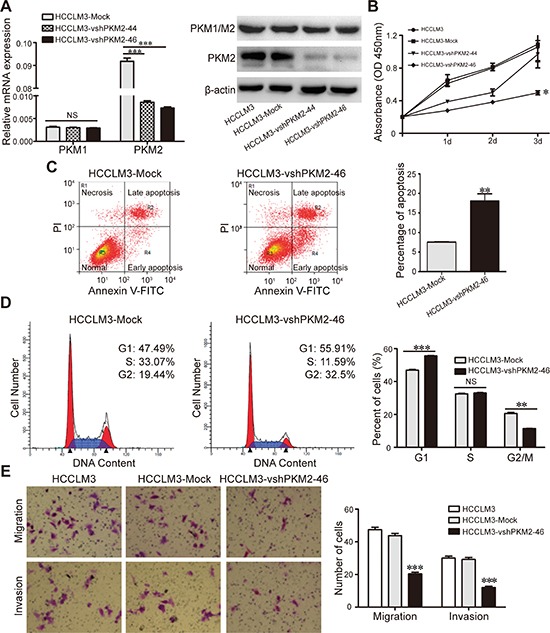
Effect of PKM2 gene suppression on HCCLM3 HCC cell lines **(A)** PKM2 expression in HCCLM3 was modified by vshRNA interference and was verified by qRT-PCR and western blot. **(B)** Cell proliferation was detected by CCK-8 assay. **(C)** The apoptosis of cancer cells were analyzed by flow cytometry for annexin-V/PI assay. **(D)** Cell cycles of cancer cells were analyzed by flow cytometry for PI staining. **(E)** The migration and invasion of cancer cells was measured by transwell assays. The data represent the mean ± SD of three different experiments. *P < 0.05. **P < 0.01. ***P < 0.001.

Because the PKM2 levels were higher in highly metastatic HCC cell lines, we used shRNA to generate PKM2-knockdown (PKM2-KD) cells to investigate the function of PKM2. As shown in Fig. 2A, PKM2 shRNA treatment led to a similar decrease in PKM2 expression at the mRNA and protein levels. We then focused on one representative shRNA to simplify the interpretation, and we explored the effects of PKM2 downregulation on cell growth using the HCCLM3 cell line. VshPKM2-46, but not vshPKM2-44, suppressed the growth of HCCLM3 cells, suggesting that PKM2 is essential for the growth of HCC cell lines (*P* < 0.05, Fig. 2B).

Next, the apoptosis and cell cycle assays revealed that PKM2 knockdown induced cellular apoptosis (18% ± 1.9% versus 7.5% ± 0.1% in the control group, *P* < 0.01; Fig. 2C) and that the cell cycle was arrested in the G1 phase, with 55.6% of the HCCLM3-vshPKM2-46 cells in G0/G1 phase versus 46.9% of the control cells (*P* < 0.001, Fig. 2D).

We then explored whether PKM2 was associated with altered cell migration and invasiveness using Boyden chamber assays. *In vitro* migration assays showed that the number of migrated HCCLM3-Mock cells was 43.8 ± 3.1, which was significantly higher than that of HCCLM3-vshPKM2-46 cells (20.4 ± 2.2, *P* < 0.001). In the *in vitro* invasion assays, the number of invasive HCCLM3-Mock cells was 29.2 ± 2.9, which was significantly higher than that of HCCLM3-vshPKM2-46 cells (12 ± 1.9, *P* < 0.001) (Fig. 2E).

Using a transmission electron microscope, we further analyzed the numbers of autophagosome-like vacuoles with double-membrane structures and found that HCCLM3-vshPKM2-46 cells contained significantly fewer of these vacuoles compared to HCCLM3 and HCCLM3-Mock cells. As shown in Fig. S1A, morphologic analysis of HCCLM3-vshPKM2-46 cells by transmission electron microscopy revealed the presence of fewer double-membrane vacuolar structures with the morphologic features of autophagosomes.

We next studied the vascular channel formation ability of different cell culture supernatants. HCCLM3-vshPKM2-46 cell supernatant suppressed the formation of tubular networks in HUVECs, in terms of number, length, and intersections, to a greater extent than HCCLM3 and HCCLM3-Mock cell supernatants. The tubule number, number of intersecting nodes, and tubule length of the HCCLM3-Mock supernatant were 31.3 ± 9, 36.7 ± 5.5, and 35.7 ± 4.2 mm, respectively, which were significantly higher than those of the HCCLM3-vshPKM2-46 supernatant (15.3 ± 1.5 (*P* < 0.05), 18.7 ± 2 (*P* < 0.001), and 12 ± 3 mm (*P* < 0.001, Fig. S1B).

To further illustrate the role of PKM2 in tumor progression, we successfully overexpressed PKM2 gene in Hep3B cells with low PKM2 expression background (Fig. S2A, S2B). As shown in Fig. S2C, the proliferation ability of Hep3B-PKM2 cells were higher than Hep3B-Mock cells (*P* < 0.001). In the *in vitro* migration assays, the number of migrated Hep3B-PKM2 cells was 44.6 ± 5.7, which was significantly higher than that of Hep3B-Mock cells (23.4 ± 7.3) (*P* < 0.01). Accordingly, *in vitro* invasion assays showed that the number of invasive Hep3B-PKM2 cells was 34.0 ± 6.3, which was higher than that of Hep3B-Mock cells (13.8 ± 4.4, *P* < 0.001) (Fig. S2D).

### PKM2 knockdown inhibits the tumor progression of Hcc *in vivo*

**Figure 3:**
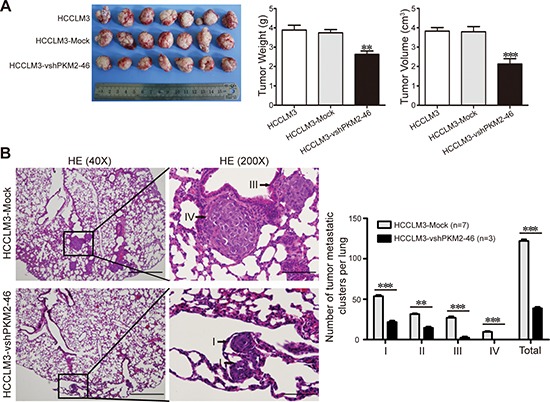
PKM2 promotes HCC progression in a xenograft nude mice model **(A)** Liver bearing tumors formed by implanted cells with different expression levels of PKM2. Weights and tumor volume of the tumors in livers of nude mice were measured. Error bar indicates standard deviation (*n* = 7). Error bar indicates standard deviation (*n* = 7). **(B)** H&E stained images of metastatic clusters in lungs from all groups with magnification of the selected areas. Scale bar, 40×, 200 μm. 200×, 50 μm. All grades of metastatic clusters in lungs of all xenograft nude mice groups. **P < 0.01. ***P < 0.001.

All cell lines successfully formed liver tumors after orthotropic transplantation into nude mice. The tumor weights of HCCLM3 and HCCLM3-Mock-derived xenografts were 3.89 ± 0.65 g and 3.74 ± 0.44 g, respectively, which were significantly larger than those of xenografts originating from HCCLM3-vshPKM2-46 cells (2.63 ± 0.47 g). Similar to the tumor weight, the tumor sizes of HCCLM3 and HCCLM3-Mock-derived xenografts were 3.83 ± 0.48 cm^3^ and 3.80 ± 0.71 cm^3^, respectively, which were significantly larger than those of xenografts originating from HCCLM3-vshPKM2-46 cells (2.13 ± 0.74 cm^3^) (Fig. 3A). Furthermore, the incidence of lung metastasis for HCCLM3-Mock and HCCLM3-vshPKM2-46 cells was 100% and 42.9%, respectively. The number of metastatic clusters in the HCCLM3-vshPKM2-46 group was much lower than that observed for the control group (*P* < 0.001, Fig. 3B).

### PKM2 mediates Mdsc infiltration *in vivo*

**Figure 4:**
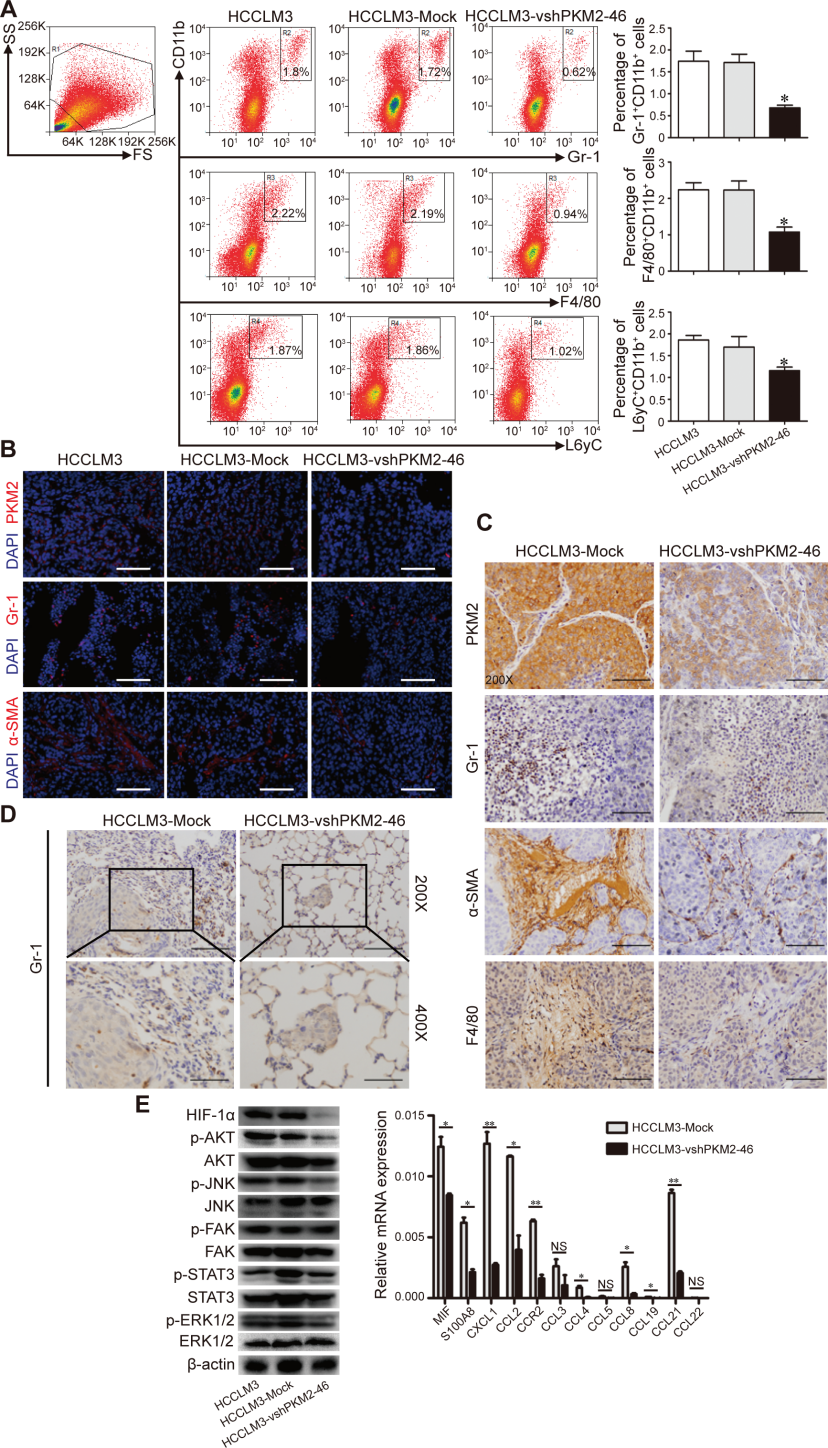
PKM2 induces MDSC infiltration in a xenograft nude mice model **(A)** Flow cytometry analysis with Gr-1, CD11b, F4/80, L6yC in different group. The percentages of Gr-1^+^CD11b^+^, F4/80^+^CD11b^+^, and L6yC^+^ CD11b^+^ cells were calculated. **(B)** Representative images from tumor serial sections stained with PKM2, Gr-1, and α-SMA by immunofluorescence. Scale bar, 100×, 100 μm. **(C)** Representative images from tumor serial sections stained with PKM2, Gr-1, α-SMA, and F4/80 by immunohistochemistry. **(D)** Representative images from lung serial sections stained with Gr-1 by immunohistochemistry. **(E)** PKM2 induces phosphorylation and activates inflammatory cytokine production in HCC cell lines. Scale bar, 200×, 50 μm. 400×, 25 μm. *P < 0.05.

In flow cytometry analysis, we found significantly decreased intratumoral percentages of Gr-1^+^CD11b^+^ granulocytic MDSCs, F4/80^+^ CD11b^+^ macrophages, and Ly6C^+^ CD11b^+^ monocytic MDSCs in the HCCLM3-vshPKM2-46-derived xenografts compared to HCCLM3- and HCCLM3-Mock-derived xenografts (Fig. 4A). In agreement with these cytometry findings, the intratumoral expression of PKM2, Gr-1, and α-SMA (α-smooth muscle actin) in HCCLM3-vshPKM2-46-derived xenografts was markedly lower than that observed in HCCLM3- and HCCLM3-Mock-derived xenografts by immunofluorescence analysis (Fig. 4B). Similar results were observed in immunohistochemistry assays (Fig 4C). Moreover, there was more extensive peritumoral and intratumoral Gr-1^+^ MDSC infiltration in metastatic nodules in the lungs of xenograft nude mice (Fig. 4D). For nodules showing a similar size between the groups, there were less Gr-1^+^ cells observed in the HCCLM3-vshPKM2-46 group compared to the HCCLM3-Mock group (Fig. S3A).

We next investigated the influence of PKM2-mediated Gr-1^+^ MDSC recruitment and premetastatic niche formation on the growth of pulmonary metastatic foci from HCCLM3 tumors. Consistent with our findings in primary HCC cells, fewer Gr-1^+^ cells were observed in the lungs of the HCCLM3-vshPKM2-46 group compared to the HCCLM3-Mock group (Fig. S3B).

### PKM2 activates the PI3K-Akt and JNK signaling pathways and HIF-1α in HCC cells

To investigate which signaling pathways associated with PKM2 contribute to HCC tumorigenesis, stably transfected cells and parent cells were used to evaluate the activation state of multiple pathways. PKM2 did not affect FAK, STAT3, or ERK1/2 phosphorylation in HCC cells. Interestingly, the downregulation of PKM2 by shRNA in HCCLM3 cells induced a significant decrease in the phosphorylation levels of Akt and JNK. Moreover, the downregulation of PKM2 by shRNA in HCCLM3 cells led to a significant decrease in HIF-1α protein (Fig. 4E).

### PKM2 knockdown alters the cytokine secretion pattern of HCC cells

Given that PKM2 knockdown resulted in reduced inflammatory leukocyte recruitment, we hypothesized that PKM2 knockdown may alter cytokine production in HCC cells. As illustrated in Fig. 4E, CCL8, CXCL1, CCL21, CCL2, and MIF were the cytokines showing the greatest decrease in comparison to control cells (87.3%, 78.3%, 76.2%, 65.9%, and 32%, respectively). CCL8 and CCL2 secreted by tumor cells were reported to recruit monocytic cells [20] [21], and CXCL1 and MIF were linked to the recruitment of MDSCs [22] [23]. To further define the role of secreted cytokine in MDSC recruitment, we performed *in vitro* transwell assays using freshly harvested MDSC [24]. The number of migrated HCCLM3-Mock-CM group was 196.6 ± 20.0, which was markedly higher than that of HCCLM3-vshPKM2-46-CM group (120.4 ± 14.5) (*P* < 0.001) (Fig. S4A). Meanwhile, the number of migrated MDSCs in Hep3B-PKM2-CM group was 154.2 ± 30.8, which was significantly higher than that of Hep3B-Mock group (43.4 ± 23.5) (*P* < 0.001). The blockade of CXCL1 and MIF significantly ameliorated this effect (Fig. S4B). These results showed that CXCL1 and MIF may participate in the PKM2-mediated recruitment of MDSC. Therefore, our data suggest that PKM2 contributes to tumor progression by modulating both tumor cells and the tumor microenvironment.

### PKM2 expression correlates with poor prognosis in HCC patients

**Figure 5:**
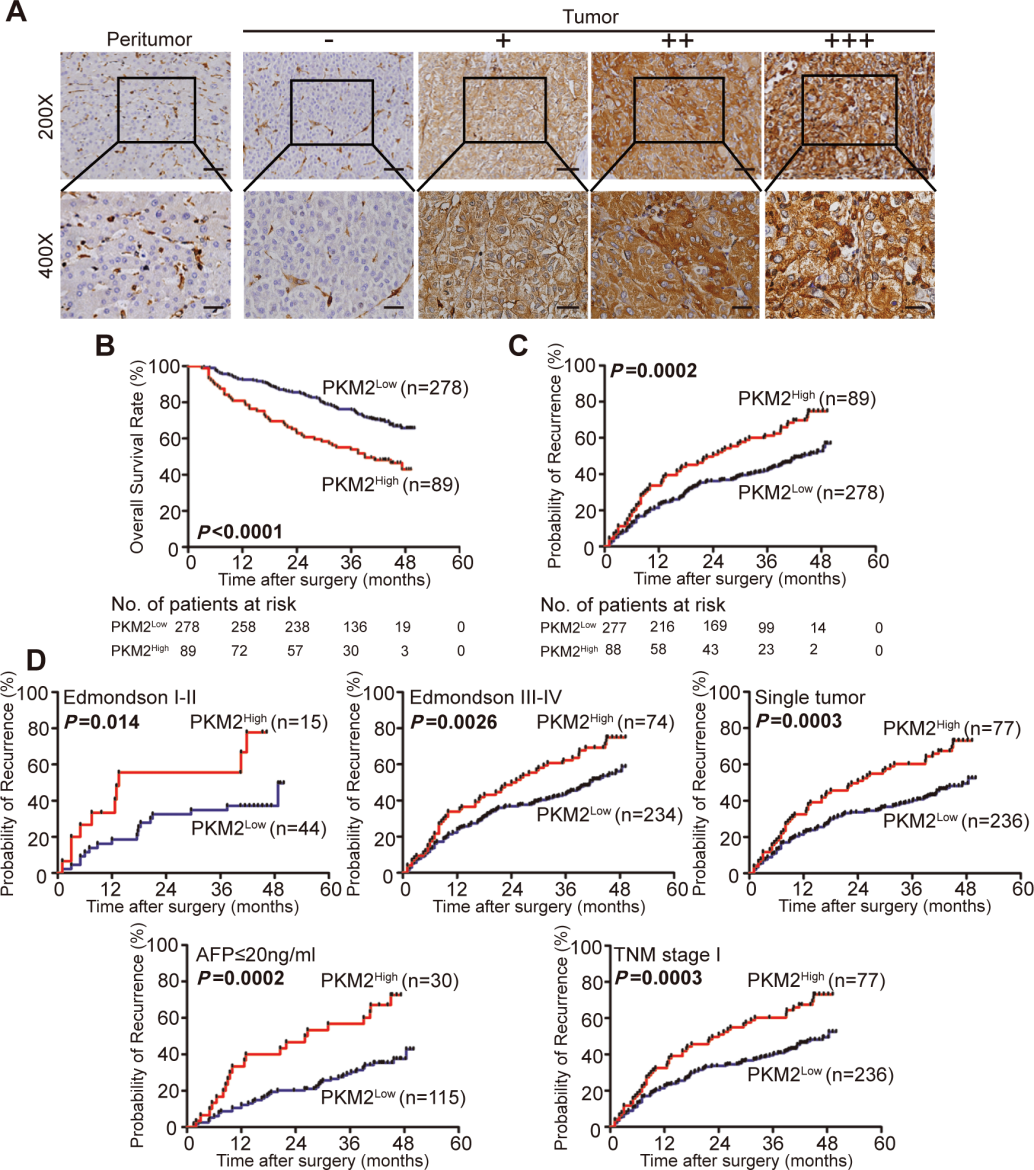
PKM2 expression and prognostic value in HCC tissue (training cohort, *n* = 367) **(A)** Representative photomicrographs of tumor tissues of the different staining patterns are presented here and are graded from 0 to +++, images of representative staining are shown. Scale bar, 200×, 50 μm. 400×, 25 μm. **(B, C)** Kaplan-Meier analysis of OS and TTR for PKM2 expression. **(D)** Prognostic role of PKM2 in Edmonson I-II, Edmonson III-IV, Single tumor, AF *P* < 20 ng/mL, and TNM stage I subgroups.

From the training cohort, representative immunohistochemical staining results are shown in Fig. 5A. We found that PKM2 expression was significantly correlated with tumor size (*P* = 0.041), microvascular invasion (*P* = 0.001), and tumor differentiation (*P* < 0.001). Other clinical characteristics, including age, sex, preoperative serum alpha-fetoprotein, γ-GT, liver cirrhosis, tumor encapsulation, and TNM stage, were not directly related to the expression of PKM2 (Table 1).

**Table 1:** Relationship Between PKM2 and Clinicopathological Features (Training Cohort)

Clinicopathological indexes		PKM2		
		Low	High	p^†^
Age(year)	≤50	110	45	0.068
	>50	168	44	
Sex	Female	39	9	0.340
	Male	239	80	
HBsAg	Negative	57	15	0.450
	Positive	221	74	
HCV	Negative	275	87	0.408
	Positive	3	2	
AFP	≤20	115	30	0.198
	>20	163	59	
γ-GT(U/L)	≤54	122	45	0.271
	>54	156	44	
Liver cirrhosis	No	44	15	0.818
	Yes	234	74	
Tumor size(cm)	≤5	192	51	0.041
	>5	86	38	
Microvascular invasion	absence	211	52	0.001
	present	67	37	
Tumor encapsulation	complete	146	40	0.214
	none	132	49	
Tumor differentiation	I + II	216	49	<0.001
	III + IV	62	40	
TNM stage	I	236	77	0.706
	II + III	42	12	

**Abbreviations:** HBsAg, hepatitis B surface antigen; AFP, α-fetoprotein; γ-GT, γ-glutamyl transferase; TNM, tumor-nodes-metastasis.

† A *p*-value < 0.05 was considered statistically significant. *p*-values were calculated using the Pearson chi-square test.

At the final follow-up in December 2011, 55.3% (203/367) of the patients suffered recurrence, and 37.1% (136/367) died. In the training cohort, the 1- and 3-year OS rates were 80% and 59%, and the 1- and 3-year cumulative recurrence rates were 39% and 60%, respectively. Moreover, we found that the 1- and 3-year survival rates of the PKM2^low^ patients were significantly higher than those of the PKM2^high^ group (86% versus 64%, and 64% versus 42%, respectively; Fig. 5B). Similarly, PKM2^high^ HCC patients demonstrated poorer prognosis at 1 and 3 years, with higher cumulative recurrence rates than PKM2^low^ patients (50% versus 36%, and 78% versus 55%, respectively; Fig. 5C).

We further investigated the predictive value of PKM2 expression within patient subgroups and found that the prognostic significance of PKM2 retained within the Edmondson I-II, Edmondson III-IV, single tumor, AFP ≤ 20 ng/mL, and TNM stage I subgroups (Fig. 5D).

In the univariate analysis, AFP, γ-GT, tumor size, microvascular invasion, and tumor differentiation were associated with OS, whereas HBsAg, AFP, γ-GT, tumor size, microvascular invasion, tumor differentiation, and TNM stage were associated with cumulative recurrence. In the multivariate analysis, tumor size and microvascular invasion were associated with OS, whereas HBsAg, AFP, tumor size, and microvascular invasion were associated with cumulative recurrence. Univariate and multivariate analyses showed that PKM2 expression in tumor cells served as an independent risk factor for both OS (*P* < 0.001, HR = 2.117; *P* = 0.001, HR = 1.901, respectively) and TTR (*P* < 0.001, HR = 1.742; *P* = 0.018, HR = 1.451, respectively; Table 2).

**Table 2:** Univariate and Multivariate Analyses of Factors Associated With Survival and Recurrence of Training Cohort

Variable	OS		TTR	
	HR (95% CI)	*p*	HR (95% CI)	*p*
**Univariate analysis**				
Age, years (≤50 vs. >50)	1.027 (0.729–1.446)	0.881	1.128 (0.851–1.497)	0.402
Sex (female vs. male)	1.055 (0.635–1.754)	0.836	0.964 (0.643–1.446)	0.860
HBsAg (negative vs. positive)	1.387 (0.879–2.189)	0.160	1.876 (1.251–2.815)	0.002
AFP, ng/ml (≤20 vs. >20)	1.601 (1.113–2.304)	0.011	1.844 (1.367–2.487)	<0.001
γ-GT, U/L (≤54 vs. >54)	1.703 (1.199–2.418)	0.003	1.417 (1.071–1.874)	0.015
Liver cirrhosis (no vs. yes)	1.116 (0.695–1.795)	0.649	1.323 (0.882–1.984)	0.177
Tumor size, cm (≤5 vs. >5)	3.220 (2.295–4.518)	<0.001	2.521 (1.907-3.333)	<0.001
Microvascular invasion (no vs. yes)	2.083 (1.478–2.935)	<0.001	2.197 (1.655–2.918)	<0.001
Tumor encapsulation (complete vs. none)	1.318 (0.940–1.846)	0.109	1.313 (0.997–1.730)	0.053
Tumor differentiation (I–II vs. III–IV)	1.560 (1.093–2.226)	0.014	1.579 (1.180–2.112)	0.002
TNM stage (I vs. II III)	1.467 (0.956–2.249)	0.079	1.496 (1.054–2.123)	0.024
PKM2 (low vs. high)	2.117 (1.489–3.011)	<0.001	1.742 (1.295–2.344)	<0.001
**Multivariate analysis**				
HBsAg (negative vs. positive)	NA	NA	1.969(1.306–2.967)	0.001
AFP, ng/ml (≤20 vs. >20)	1.386 (0.961–2.000)	0.080	1.604 (1.184–2.173)	0.002
γ-GT, U/L (≤54 vs. >54)	1.350 (0.934–1.952)	0.111	1.187 (0.888–1.587)	0.247
Tumor size, cm (≤5 vs. >5)	2.718 (1.895–3.900)	<0.001	2.298 (1.708–3.093)	<0.001
Microvascular invasion (no vs. yes)	1.472 (1.030–2.105)	0.034	1.545 (1.148–2.078)	0.004
Tumor differentiation (I–II vs. III–IV)	1.256 (0.869–1.815)	0.224	1.275 (0.942v1.724)	0.116
TNM stage (I vs. II III)	NA	NA	1.487 (1.040–2.126)	0.030
PKM2 (low vs. high)	1.901 (1.321–2.737)	0.001	1.451 (1.067–1.973)	0.018

**Abbreviations:** TTR, time to recurrence; OS, overall survival; AFP, α-fetoprotein; γ-GT, γ-glutamyl transferase; TNM, tumor-nodes-metastasis; HR, hazard ratio; CI, confidential interval; NA, not adopted; NS, not significant.

^†^Cox proportional hazards regression.

### Independent validation

The prognostic value of PKM2 protein expression was validated in an independent validation cohort of 354 HCC patients by immunohistochemical staining (Table S2; Fig. S5A–D). In accordance with the abovementioned findings, univariate and multivariate analyses revealed that PKM2 served as an independent risk factor for both OS (*P* < 0.001, HR = 2.277; *P* = 0.027, HR = 1.572, respectively) and TTR (*P* < 0.001, HR = 2.054; P = 0.01, HR = 1.593, respectively; Table S3).

## DISCUSSION

PKM2 has been shown to play a dominant role not only in cancer metabolism but also in the direct regulation of gene expression and subsequent cell cycle progression [25, 26]. PKM2 was expressed predominantly in tumor cells and was important for tumor growth *in vitro* and *in vivo*, as targeting PKM2 expression by siRNA or shRNA inhibited tumor proliferation and induced apoptosis [5, 27]. Immunohistochemical results further showed that PKM2 held diagnostic value for various cancers [28–30]. Several studies have detected high serum levels of PKM2 in many different types of tumors [31, 32]. However, the relationship between PKM2 and HCC remains limited; to the best of our knowledge, only one study has shown that the PKM2 mRNA level was positively associated with proliferative activity [13]. Therefore, our study was the first to evaluate the expression and biological function of PKM2 in HCC, and our findings revealed the putative mechanism of PKM2 in knockdown experiments and the prognostic value of PKM2 in HCC patients.

The current results show that PKM2 expression was markedly upregulated in HCC tissues in comparison to adjacent non-tumor liver tissues. Our analysis further illuminated the role of PKM2 deregulation using *in vitro* and *in vivo* assays. We discovered that PKM2 downregulation suppressed HCC cell proliferation and invasion *in vitro*, induced apoptosis and G1 arrest, and restrained *in vivo* tumor growth and metastasis. These results indicate that PKM2 may represent a novel oncogene that plays central roles in the regulation of tumor growth and metastasis in HCC.

Autophagy has contradictory functions in cancer. It can suppress tumors through the elimination of oncogenic protein substrates, or, in some circumstances, it can promote tumors through the autophagy-mediated intracellular recycling of metabolic substrates [33]. In line with our previous findings that autophagy plays a tumor-promoting role in HCC [34–36], we found that PKM2 knockdown inhibited autophagy vehicle formation. One possibility is that PKM2 participated in a positive feedback loop that promoted HIF-1α transactivation [11], as HIF-1α was a known activator of autophagy [37]. In this study, we found that the downregulation of PKM2 by shRNA treatment in HCCLM3 cells led to a significant decrease in HIF-1α. Chiavarina B et al also found that PKM2 overexpression in normal human fibroblasts induced an NF-κB-dependent autophagic program [38]. But on the other side, PKM2 knockdown decreased AKT phosphorylation may result in downstream mTOR inhibition which may expectedly lead to autophagy activation. However, whether this finding may be due to mTOR-independent mechanisms and what will happen under starvation conditions need to be further illustrated.

Angiogenesis provides tumors with nutrients and evacuates metabolic waste and carbon dioxide. Our data show that PKM2 knockdown led to reduced vascular channel formation ability, with reductions in tubule number, intersecting nodes, and tubule length. One possible reason for this result may be related to the role of PKM2 as a transactivator for HIF, as HIF-1α was proposed to contribute to angiogenesis [39]. However, future studies should further explore the mechanisms responsible for this relationship between PKM2 and HIF-1α.

Increasing connections between inflammation and cancer have recently been identified. By supplying bioactive molecules to the tumor microenvironment, tumor-associated inflammation contributes to multiple tumor characteristics [2]. HCC development was also accompanied by ongoing inflammation caused by cellular infiltration. The HCC milieu was characterized by tumor-associated macrophages (TAMs) and MDSCs [40], which exerted their immunosuppressive function by inducing regulatory T cells and suppressing the function of NK cells [24, 41]. In our study, we found that PKM2 promoted HCC progression in a nude mouse xenograft model by recruiting Gr-1^+^CD11b^+^ granulocytic MDSCs, F4/80^+^ CD11b^+^ macrophages, and Ly6C^+^ CD11b^+^ monocytic MDSCs to the tumor microenvironment. Moreover, myofibroblasts, which can be identified by α-SMA expression and constitute the predominant cell population within the tumor stroma, were downregulated upon PKM2 knockdown in our nude mouse xenograft model. To address the mechanism for the inflammatory recruitment role of PKM2, we observed the induction of downstream chemokines such as CCL8, CCL2, CXCL1, and MIF, which recruit MDSCs and TAMs, respectively. Thus, we suggest that PKM2 modulates the tumor microenvironment by secreting cytokines and recruiting inflammatory cells such as MDSCs and TAMs.

Circulating levels of MDSCs were increased in the bone marrow, peripheral blood, and spleens of tumor-bearing mice compared with non-tumor-bearing-mice, and MDSC recruitment played an important role in premetastatic niche formation leading to the growth of pulmonary metastatic foci [42]. In this study, we not only observed greater intratumoral MDSC infiltration but also greater pulmonary MDSC infiltration in the lungs of control nude mice versus shPKM2 knockdown-treated nude mice. Thus, PKM2 plays an essential role in mediating MDSC migration to premetastatic organs, and CXCL1 and MIF may participate in the PKM2-mediated recruitment of MDSCs.

To investigate whether PKM2 was associated with the clinical outcome of HCC patients after curative resection, we analyzed the PKM2 expression in TMAs obtained from two cohorts (367 HCC cases and 354 HCC cases, respectively) using immunohistochemistry. We found that PKM2 expression in HCC tissues was inversely correlated with patients' OS and TTR. Furthermore, there was a significant positive correlation between PKM2 expression and tumor size, microvascular invasion, and tumor differentiation. Therefore, our results suggest a possible oncogenic role for PKM2 in HCC.

Our study has limitations. On one hand, we mainly focused on the efficiency of PKM2 on innate immunity; the efficiency of PKM2 on adaptive immunity will be investigated in a future study. On the other hand, we found only indirect evidence of a relationship between PKM2 and paracrine cytokines; thus, direct evidence of such a relationship should be illustrated in future experiments, such as those using a chromatin immunoprecipitation (ChIP) assay. Finally, a major drawback of the present study was its retrospective nature. Subsequent therapies, such as radiofrequency ablation (RFA), percutaneous ethanol injection (PEI), transcatheter arterial chemoembolization (TACE), or external radiotherapy for patients with HCC recurrence, might influence the OS results.

In conclusion, PKM2 promotes HCC cell proliferation, migration, resistance to apoptosis, angiogenesis, autophagy, intratumoral inflammatory cell infiltration, and premetastatic niche formation. Moreover, PKM2 expression may serve as a novel prognostic indicator for HCC patients after curative resection, and these findings therefore have important implications for designing immunotherapies targeting PKM2.

## MATERIALS AND METHODS

### Cell lines

The human HCC cell lines MHCC97L, MHCC97H, and HCCLM3, which have the same genetic background but different lung metastatic potentials, were established at our institute [43] [44]. Normal L02 hepatocyte cells and the HCC cell lines Hep3B, HepG2, SMMC-7721, and Huh7 were purchased from the Institute of Biochemistry and Cell Biology, Chinese Academy of Science. All cell lines were routinely maintained and cultured.

### Patients and follow-up

Forty-eight sets of fresh, matching tumor and peritumor tissue samples, which were used for qRT-PCR analysis, were randomly collected from HCC patients who underwent curative resection with informed consent between 2009 and 2010 at the Liver Cancer Institute, Zhongshan Hospital, Fudan University, Shanghai, China. Investigation has been conducted in accordance with the ethical standards and according to the Declaration of Helsinki and according to national and international guidelines and has been approved by the institutional review board the Liver Cancer Institute. All tissues were collected immediately upon resection of the tumors in the operation theater, transported in liquid nitrogen, and then stored at –80°C.

### RNA isolation and qRT-PCR

Total RNA was isolated from different cell lines using the RNeasy Mini Kit (Qiagen, Valencia, CA) according to the manufacturer's instructions. Equal amounts of RNA were reverse transcribed into cDNA using the SuperScript III System (Invitrogen, Carlsbad, CA). PKM2 mRNA expression in the HCC cell lines and tumor tissues from 48 HCC patients was measured by qRT-PCR using an ABI7900 HT instrument (Applied Biosystems, Foster, Foster City, CA). qRT-PCR was performed using the SYBR PrimeScript RT-PCR Kit (TAKARA BIO INC., Otsu, Shiga, Japan) in accordance with the manufacturer's instructions. β-actin was used as an internal control. The mRNA levels were calculated based on the Ct values, which were corrected for β-actin expression according to the following equation: 2-^ΔΔCt^ [ΔCt = Ct (PKM2) – Ct (β-actin)]. All experiments were performed in triplicate. The primers used for all genes are listed in Table S4.

### Western blotting and immunofluorescence staining

Whole cell lysates were harvested using cell lysis buffer supplemented with a protease inhibitor cocktail (Sigma-Aldrich, St. Louis, MO). The nuclear and cytosol extracts were isolated using the nuclear extract kit (Active Motif, Carlsbad, CA) according to the manufacturer's instructions. The signals were visualized using enhanced chemiluminescence (Pierce, Rockford, IL, USA). The antibodies used are listed in Table S5. For the immunofluorescence assays, the cells were fixed with 4% formaldehyde and then permeabilized with PBS containing 0.2% Triton X-100. The slides were blocked using 5% BSA and incubated with anti-PKM2 antibody at room temperature for 1 h. The cells were treated with the primary antibody overnight at 4°C. Then, the cells were incubated with 2 μg/mL Alexa Fluor 594^®^ phalloidin (Invitrogen, Carlsbad, CA) for 30 min at room temperature. The slides were washed with PBS, mounted in DAPI, and observed by fluorescence microscopy (Leica Microsystems Imaging Solutions, Cambridge, UK). As a negative control, the primary specific antibodies were replaced with control mouse IgG.

### Cell transfection and stable clone selection

ShRNA constructs in pLKO.1-puro specifically targeting the human and PKM2 sequences and pLenti6/V5-D-TOPO harboring the full-length of PKM2 gene were purchased from GenePharma Co. The shRNA target sequences were as follows: shRNA_44: AGGCAGAGGCUGCCAUCUA. shRNA_46: GCCAUAAUCGUCCUCACCA. The lentiviral vector and plasmid were transfected into cells according to the manufacturer's instructions. Control cells were transfected with a control shRNA that did not match any known human coding cDNA. Stable knockdown clones were pooled and used for the experiments. Stably transfected clones were validated by immunoblotting for PKM2.

### Cell proliferation assay and cell matrigel migration and invasion assays

Cells were seeded in 100 μL aliquots into 96-well plates. At the indicated time points, 10 μL CCK-8 solution (Dojindo, Kumamoto, Japan) was added to the cells, and the plates were incubated for an additional 2 h. The absorbance at 450 nm was measured to determine the number of viable cells in each well. All experiments were performed in triplicate.

For the Matrigel migration and invasion assays, cell culture was performed using Transwell chambers (8 μm, 24-well format; Corning, USA). For the invasion assay, the insert membranes were coated with diluted Matrigel (BD Biosciences, BD Biosciences, San Jose, CA). Cells (1 × 10^5^) were then added to the upper chamber and cultured for 48 h. For the migration assay, the insert membranes were not coated with Matrigel (BD Biosciences, San Jose, CA), and the cells were cultured in the same conditions. Finally, the insert membranes were cut and stained with crystal violet (0.04% in water; 100 μL), and the permeating cells were counted under an inverted microscope. All experiments were performed in triplicate.

Another migration assay was examined *in vitro* using Transwell chambers (3 μm, 24-well format; Corning, USA) as the manufacturer's instructions. CD14^+^HLA-DR^−^ MDSCs were sorted as described [24]. Then cells were serum deprived for 24 h, then 2.5 × 10^4^ cells were seeded in triplicate on Matrigel-uncoated inserts, moved to chambers containing different conditioned medium as a chemoattractant and incubated at 37°C for 24 h. We placed different CM (conditioned medium) from HCCLM3-Mock-CM, HCCLM3-vshPKM2-46, Hep3B-CM, Hep3B-PKM2-CM, Hep3B-PKM2-CM+anti-CXCL1 (R&D System, AF275, USA, 0.15ug/mL), Hep3B-PKM2-CM+anti-MIF (4-IPP, Sigma-Aldrich, SML0472, 10 μmol/L), and Hep3B-PKM2-CM+anti-CXCL1+anti-MIF into lower transwell chambers. Five fields were randomly chosen and counted for each membrane. All experiments were performed in triplicate.

### Flow cytometry analysis

Cell cycle and apoptosis analyses were performed using Annexin V–FITC and PI staining (BD Biosciences, San Jose, CA). For cell cycle analysis, the cells were seeded in 6-well plates at 2 × 10^5^ cells per well. Forty-eight hours after transfection, the cells were fixed in 70% ethanol at 4°C for 24 h and stained with 50 μg/mL propidium iodide (PI) (BD Biosciences, San Diego, CA). The cell cycle distribution was analyzed by flow cytometry (Epics Altra, Beckman Coulter, USA). For intratumoral lymphocyte staining, xenograft tumors were mechanically dissociated to yield a single-cell solution, which was treated with ammonium chloride–potassium buffer for red blood cell lysis. The cells were treated with Fc-block (eBioscience, San Diego, CA), and surface staining was performed using CD11b PE, F4/80 APC, Gr-1 FITC, CD45 ef450, and Ly6C APC-Cy7 antibodies. For the intracellular isotype control (IgG1), κPE (eBioscience, San Diego, CA) was used. Samples were acquired and analyzed by flow cytometry (Epics Altra, Beckman Coulter, USA). The antibodies used are listed in Table S5.

### Electron microscopy

Cells were immediately fixed with 2.5% glutaraldehyde containing 0.1 mol/L sodium cacodylate and stored at 4°C until embedding. The samples were post-fixed with 1% osmium tetroxide, followed by dehydration with an increasing concentration gradient of ethanol and propylene oxide. Then, the samples were embedded in Epon 618 (Ladd Research Industries, Inc., Burlington, VT), and ultrathin (50–60 nm) sections were cut using an ultramicrotome (LKB-I). After the samples were stained with 3% uranyl acetate and lead citrate, the images were examined using a CM-120 transmission electron microscope (Philips, Holland).

### Endothelial tube formation assay

Angiogenesis *in vitro* was assessed using the endothelial tube formation assay kit (Cell Biolabs, San Diego, CA, USA). Briefly, each well of pre-chilled 96-well culture plates was coated with a thin layer of extracellular matrix gel prepared from Engelbreth-Holm-Swarm tumor cells (50 μl/well), which was left to polymerize at 37°C for 1 h. Human umbilical vein endothelial cells (HUVECs) were re-suspended in the supernatants obtained from HCCLM3, HCCLM3-Mock, and HCCLM3-vshPKM2-46 cells. HUVECs (2 × 10^4^ cells/well) were then added to the polymerized extracellular matrix gel in 300 μL of serum-free medium. After 18 h of incubation at 37°C with 5% CO_2_, the tubule numbers were assessed under a light microscope.

### *In vivo* tumorigenesis and metastasis

Athymic nude mice were purchased from the Shanghai Institute of Material Medicine and maintained in a pathogen-free environment. The animal care and experimental protocols were performed in accordance with the guidelines established by the Shanghai Medical Experimental Animal Care Commission. Ethical approval was obtained from the Zhongshan Hospital Research Ethics Committee.

HCCLM3, HCCLM3-Mock, and HCCLM3-vshPKM2-46 cells (5 × 10^6^ cells) were suspended in 100 μL serum-free Dulbecco's modified Eagle medium containing Matrigel (BD Biosciences, San Jose, CA) (1:1), and then the cells were injected subcutaneously into the upper left flank region of nude mice. When the subcutaneous tumors reached approximately 1 cm in length at 4 weeks after injection, they were removed and minced into small pieces of equal volume (1 mm^3^) and injected into the livers of 21 different nude mice (*n* = 7 for each group). Tumor growth was monitored, and the mice were sacrificed under anesthesia after 6 weeks of tumor growth. Tumor, liver, and lung tissues were removed, fixed in formalin, and embedded in paraffin. Consecutive sections were prepared for each lung tissue block and stained with hematoxylin and eosin.

The tumor volume was measured using the following formula: V = π/6× (larger diameter) × (smaller diameter) ^2^. The number of lung metastases was calculated and evaluated independently by two pathologists. Based on the number of HCC cells in the maximal section of the metastatic lesion, lung metastases were classified into four grades: grade I, ≤ 20 tumor cells; grade II, 20 to 50 tumor cells; grade III, 50 to 100 tumor cells; and grade IV, >100 tumor cells. The expression of PKM2, Gr-1, α-SMA, and F4/80 in excised tumors was examined in serial tissue sections by immunofluorescence and immunohistochemistry. The antibodies used are listed in Table S5.

### Tissue microarrays (TMAs) and immunohistochemistry

Two groups of formalin-fixed paraffin-embedded human HCC specimens were randomly collected from patients at our institute between 2006 (validation cohort, *n* = 354) and 2007 (training cohort, *n* = 367). TMAs were constructed by Shanghai Biochip Co, Ltd, as previously described [45]. The histopathological diagnosis was determined according to the World Health Organization criteria. Tumor differentiation was graded using the Edmondson grading system [46]. Tumor staging was based on the 6th edition of the tumor-node-metastasis (TNM) classification of the International Union Against Cancer. The clinicopathologic characteristics of the two cohorts are summarized in Supplementary Table S1. Follow-up data were summarized at the end of December 2011, with a median observation time of 52.2 months. Follow-up procedures and postsurgical patient surveillance were described previously [45, 47]. Overall survival (OS) was defined as the interval between the dates of surgery and death. Time to recurrence (TTR) was defined as the interval between the dates of surgery and the dates of any diagnosed recurrence (intrahepatic recurrence and extrahepatic metastasis). For surviving patients, the data were censored at the date of death or final follow-up.

Immunohistochemistry staining of paraffin sections was carried out using using a two-step protocol (Novolink Polymer Detection System, Novocastra). After antigen retrieval, the slices were incubated with the primary antibody overnight at 4°C, followed by incubation with the secondary antibody (Gene Tech, cat no. GK500705, Shanghai, China) at 37°C for 30 min. For rat-origin antibodies, a goat-anti-rat secondary antibody (Sigma-Aldrich, cat no. A5795, St. Louis, MO) was applied. The sections were stained with DAB and counterstained with hematoxylin, dehydrated in ethanol, cleared in xylene, and cover-slipped. Negative controls were included in all assays and did not receive treatment with the primary antibodies.

### Evaluation of immunohistochemical variables

Immunohistochemical scores were evaluated by three independent pathologists without knowledge of the patient characteristics, and discrepancies were resolved by discussion. The immunostains were scored using a 4-point scale (0-+++) based on the number of positive cells and the intensity of the staining. Briefly, no staining was recorded as “0”, weak staining in fewer than one-third of the cells as “+”, moderate staining in one-third to two-thirds of the cells as “++”, and strong staining in more than two-thirds of the cells as “+++”. PKM2 expression was categorized by staining intensity as low (0 and “+”) or high (“++” and “+++”). HCC patients with low or high expression were classified as PKM2^low^ or PKM2^high^, respectively. Duplicate spots for each tumor showed a good level of homogeneity for the staining protocol. The higher score was adopted as the final score in cases with differences between duplicate tissue cores.

### Statistical analysis

The preoperative clinical data, intraoperative and pathological findings, postoperative mortality, and OS and TTR rates were compared between patients with high and low expression of PKM2. The χ^2^ test or Fisher's exact test was used for quantitative variable comparison. Survival was determined using the Kaplan–Meier method, and survival curves between different groups were calculated with the log-rank test. A Cox proportional hazard regression model was used for univariate and multivariate analyses, and multivariate analysis was performed using a forward, stepwise Cox regression model. Statistical analyses were performed with SPSS 19.0 for Windows (SPSS Inc., Chicago, IL). Two-tailed *p* values < 0.05 were considered statistically significant.

## SUPPLEMENTARY FIGURES AND TABLES



## List of abbreviations

PKM2: pyruvate kinase M2
OS: overall survival
TTR: time to recurrence
HCC: hepatocellular carcinoma
qRT-PCR: quantitative reverse transcription-polymerase chain reaction
TMA: tissue microarray
shRNA: short hairpin RNA
MDSC: myeloid-derived suppressor cells
