# Supplementary material for: PKM2 promotes metastasis by recruiting myeloid-derived suppressor cells and indicates poor prognosis for hepatocellular carcinoma
Source: Oncotarget. 2014 Dec 2;6(2):846–61. doi: 10.18632/oncotarget.2749 (PMC4359260; doi:10.18632/oncotarget.2749)
Supplement: Supplementary file 1 [file oncotarget-06-846-s001.pdf]

SUPPLEMENTARY FIGURES AND TABLES

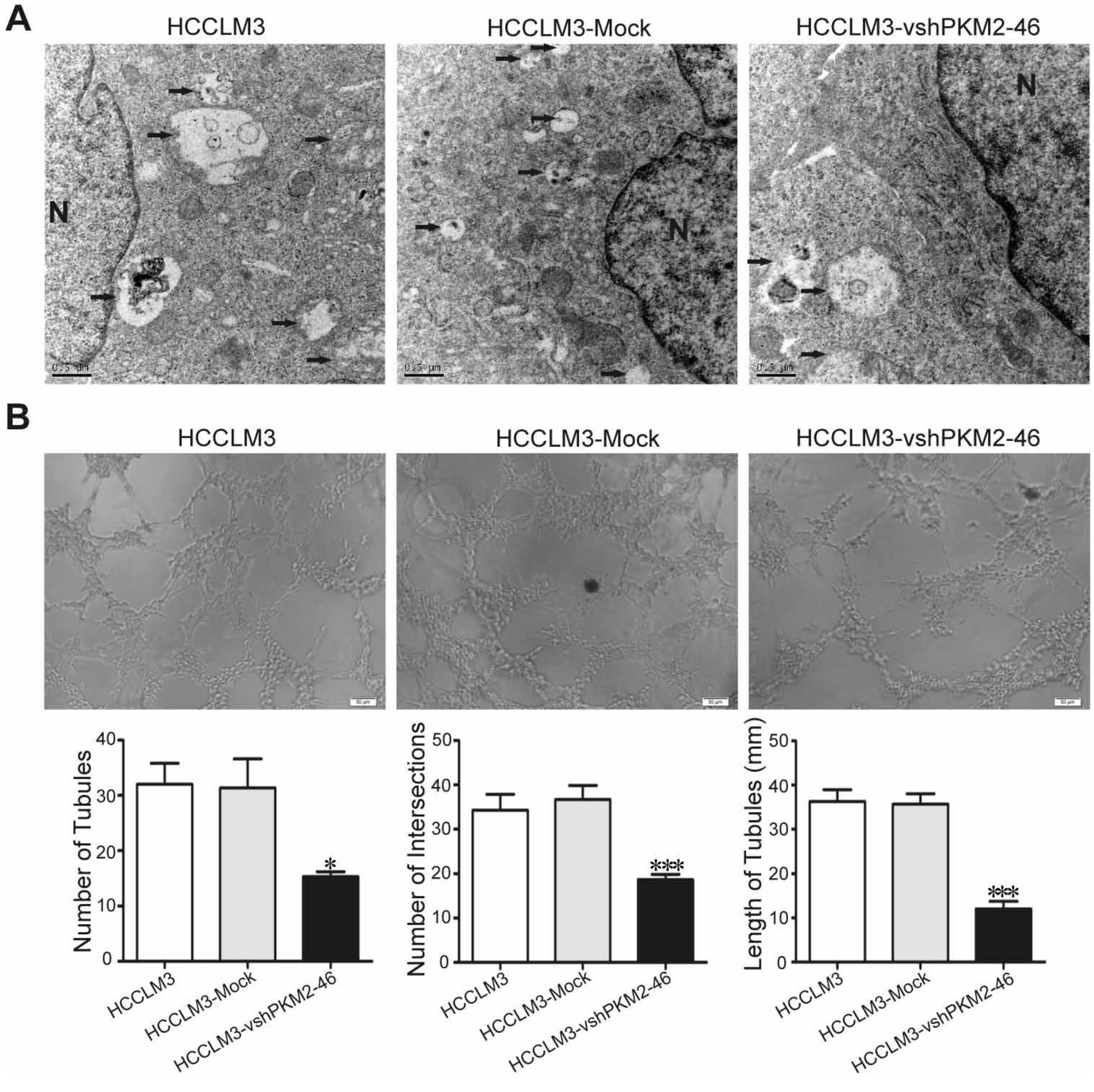

Supplementary Figure 1: Effect of PKM2 gene suppression on HCCLM3 HCC cell lines. (A) Electron microscopic analysis of different groups. N, nucleus. Scale bar, 0.5 μm. **(B)** Effects of PKM2 on tubular formation *in vitro*. Scale bar, 50 μm. Quantification of tubular formation by cancer cells. The bar charts represent the counting of tubular number, intersecting nods and tubular length between different groups. The data represent the mean ± SD of three different experiments. *$P < 0.05$. ***$P < 0.001$.

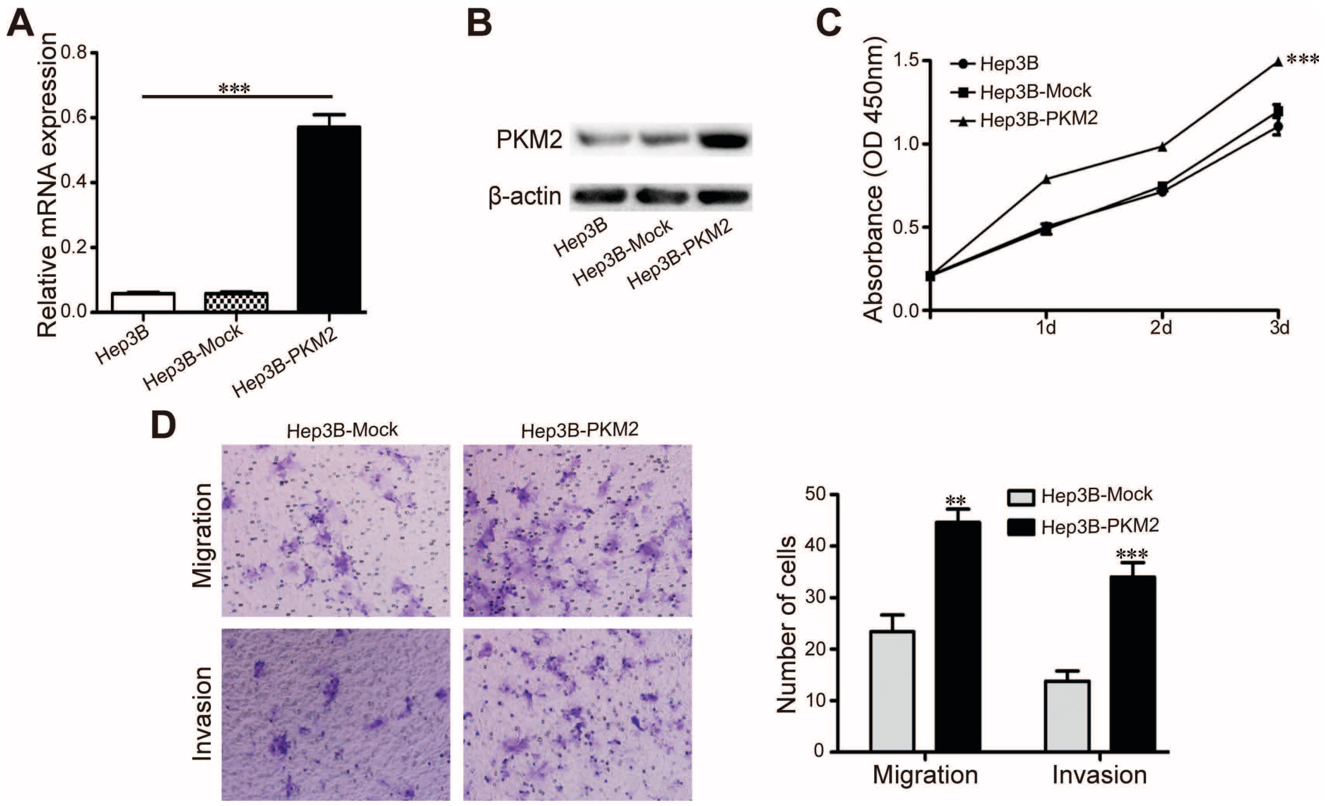

Supplementary Figure 2: Effect of PKM2 gene overexpression on Hep3B cell lines. **(A)** PKM2 overexpression in Hep3B was verified by qRT-PCR. **(B)** PKM2 overexpression in Hep3B was verified by Western Blot. **(C)** Cell proliferation was detected by CCK-8 assay. **(D)** The migration and invasion of cancer cells was measured by transwell assays. $^{**}P < 0.01$. $^{***}P < 0.001$.

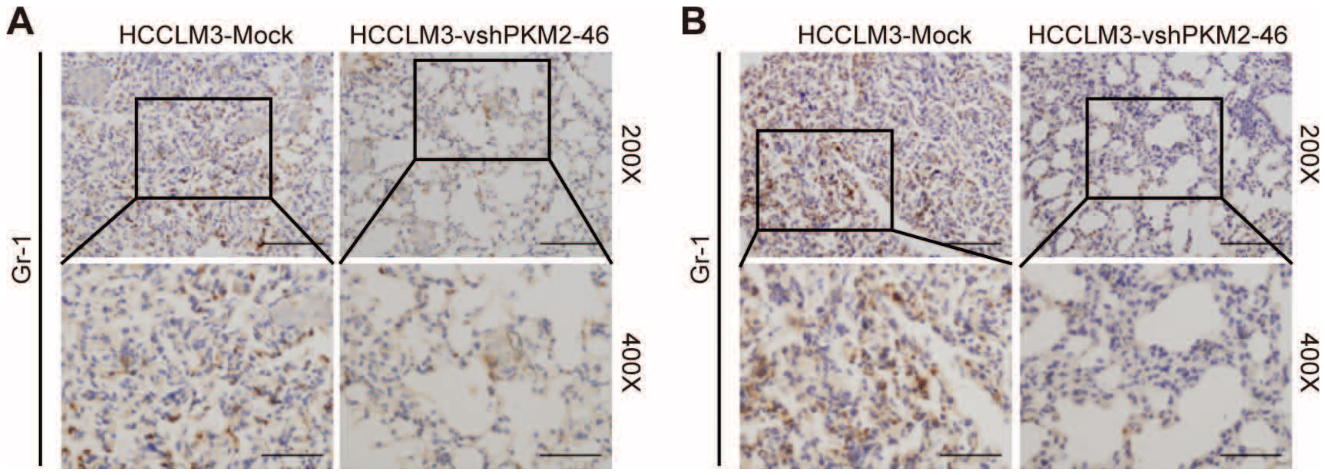

Supplementary Figure 3: MDSCs infiltration in lungs of xenograft nude mice model. **(A)** Representative images from lung serial sections with identical metastatic nodule size stained with Gr-1 by immunohistochemistry. **(B)** Representative images from lung serial sections stained with Gr-1 by immunohistochemistry. Scale bar, 200×, 50 μm. 400×, 25 μm.

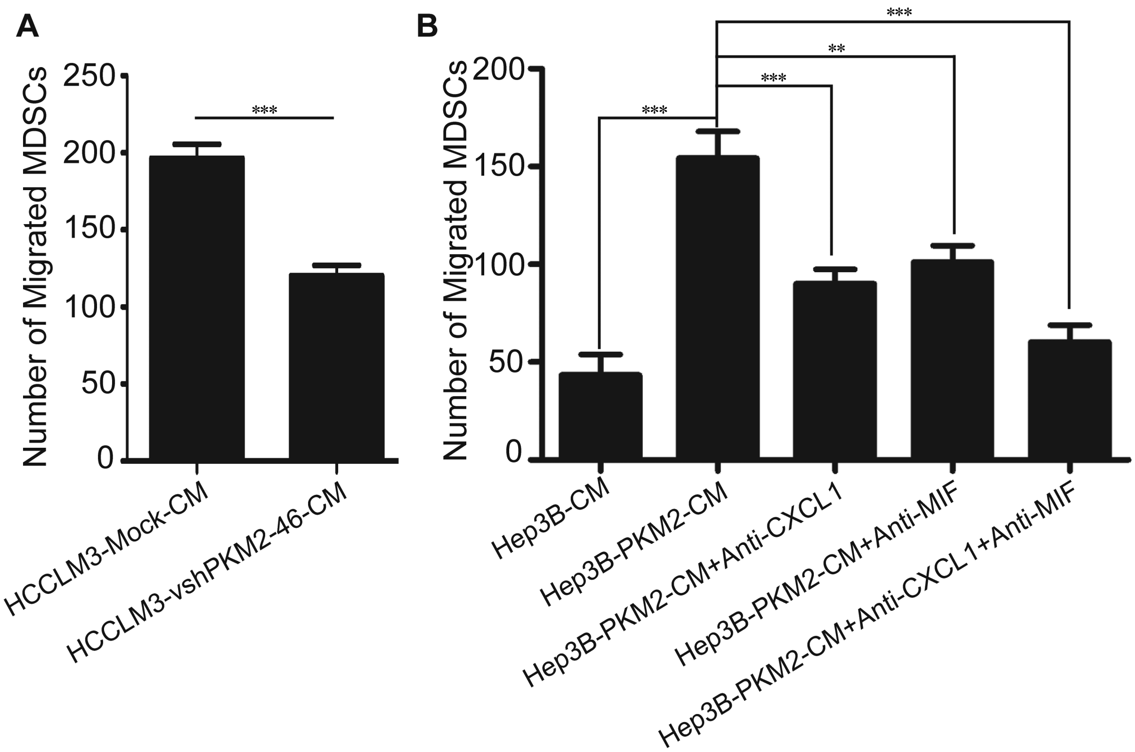

Supplementary Figure 4: The number of migrated MDSCs under different CM. $^{**}P < 0.01$. $^{***}P < 0.001$.

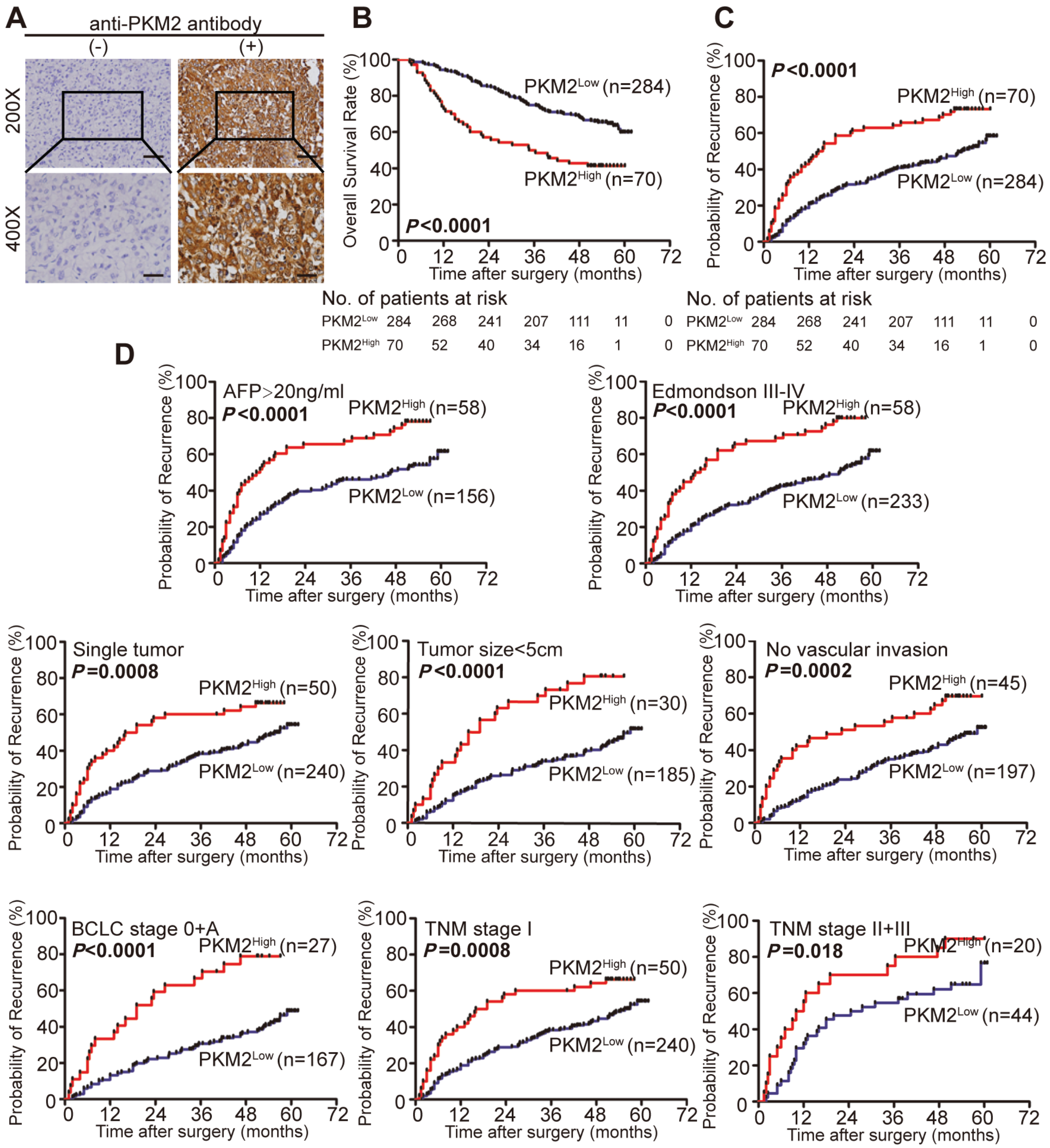

Supplementary Figure 5: PKM2 expression and prognostic value in HCC tissue (validation cohort, *n* = 354). **(A)** Representative IHC staining of human breast cancers using normal mouse IgG (left panel) and anti-PKM2 antibody (right panel) is shown. **(B, C)** Kaplan-Meier analysis of OS and TTR for PKM2 expression. **(D)** Prognostic role of PKM2 in AFP>20 ng/mL, Edmonson III–IV, Single tumor, Tumor size <5cm, No vascular invasion, BCLC stage 0 + A, TNM stage I, and TNM stage II + III subgroups. Scale bar, 200 ×, 50 µm. 400 ×, 25 µm.

Supplementary Table S1: Clinicopathologic Features of the Training Cohort and Validation Cohort

		Training Cohort (n = 367)		Validation Cohort (n = 354)	
		N	%	N	%
Age(year)	≤50	155	42.2	141	39.8
	>50	212	57.8	213	60.2
Sex	Female	48	13.1	55	15.5
	Male	319	86.9	299	84.5
HBsAg	Negative	72	19.6	54	15.3
	Positive	295	80.4	300	84.7
HCV	Negative	362	98.6	351	99.2
	Positive	5	1.4	3	.8
AFP	≤20	145	39.5	140	39.5
	>20	222	60.5	214	60.5
γ-GT(U/L)	≤54	167	45.5	182	51.4
	>54	200	54.5	172	48.6
Liver cirrhosis	No	59	16.1	63	17.8
	Yes	308	83.9	291	82.2
Tumor size(cm)	≤5	243	66.2	215	60.7
	>5	124	33.8	139	39.3
Microvascular invasion	absence	263	71.7	242	68.4
	present	104	28.3	112	31.6
Tumor encapsulation	complete	186	50.7	193	54.5
	none	181	49.3	161	45.5
Tumor differentiation	I + II	265	72.2	265	74.9
	III + IV	102	27.8	89	25.1
TNM stage	I	313	85.3	290	81.9
	II + III	54	14.7	64	18.1

Abbreviations: HBsAg, hepatitis B surface antigen; AFP, α-fetoprotein; γ-GT, γ-glutamyl transferase; TNM, tumor-nodes-metastasis.

Supplementary Table S2: Relationship Between PKM2 and Clinicopathological Features (Validation Cohort)

Clinicopathological indexes		PKM2		
		Low	High	$p^{\dagger}$
Age(year)	≤50	105	36	0.027
	>50	179	34	
Sex	Female	41	14	0.250
	Male	243	56	
HBsAg	Negative	50	4	0.013
	Positive	234	66	
HCV	Negative	282	69	0.554
	Positive	2	1	
AFP	≤20	128	12	<0.001
	>20	156	58	
γ-GT(U/L)	≤54	149	33	0.425
	>54	135	37	
Liver cirrhosis	No	51	12	0.873
	Yes	233	58	
Tumor size(cm)	≤5	185	30	0.001
	>5	99	40	
Microvascular invasion	absence	197	45	0.413
	present	87	25	
Tumor encapsulation	complete	162	31	0.055
	none	122	39	
Tumor differentiation	I + II	232	33	<0.001
	III + IV	52	37	
TNM stage	I	240	50	0.011
	II + III	44	20	

Abbreviations: HBsAg, hepatitis B surface antigen; AFP, α-fetoprotein; γ-GT, γ-glutamyl transferase; TNM, tumor-nodes-metastasis.

†A *p*-value < 0.05 was considered statistically significant. *p*-values were calculated using the Pearson chi-square test.

Supplementary Table S3: Univariate and Multivariate Analyses Of Factors Associated With Survival And Recurrence Of Validation Cohort Variable

Variable	OS		TTR	
	HR (95% CI)	$p^{\dagger}$	HR (95% CI)	$p^{\dagger}$
**Univariate analysis**				
Age, years (≤50 vs. >50)	0.963 (0.688–1.349)	0.828	1.033 (0.776–1.376)	0.822
Sex (female vs. male)	0.779 (0.509–1.192)	0.249	1.160 (0.777–1.730)	0.468
HBsAg (negative vs. positive)	1.130 (0.704–1.813)	0.614	1.147 (0.764–1.721)	0.508
AFP, ng/ml (≤20 vs. >20)	2.086 (1.447–3.008)	<0.001	1.610 (1.199–2.162)	0.002
γ-GT, U/L (≤54 vs. >54)	1.795 (1.283–2.510)	0.001	1.654 (1.248–2.192)	<0.001
Liver cirrhosis (no vs. yes)	1.504 (0.928–2.438)	0.098	1.649 (1.084–2.508)	0.020
Tumor size, cm (≤5 vs. >5)	1.904 (1.370–2.647)	<0.001	1.588 (1.199–2.101)	0.001
Microvascular invasion (no vs. yes)	1.652 (1.179–2.315)	0.004	1.651 (1.236–2.205)	0.001
Tumor encapsulation (complete vs. none)	1.327 (0.955–1.844)	0.092	1.249 (0.945–1.650)	0.119
Tumor differentiation (I-II vs. III-IV)	1.629 (1.141–2.327)	0.007	1.534 (1.129–2.085)	0.006
TNM stage (I vs. II III)	1.934 (1.333–2.807)	0.001	1.828 (1.316–2.540)	<0.001
PKM2 (low vs. high)	2.277 (1.583–3.276)	<0.001	2.054 (1.491–2.827)	<0.001
**Multivariate analysis**				
AFP, ng/ml (≤20 vs. >20)	1.642 (1.118–2.412)	0.011	1.258 (0.922–1.717)	0.147
γ-GT, U/L (≤54 vs. >54)	1.569 (1.113–2.212)	0.010	1.530 (1.148–2.040)	0.004
Liver cirrhosis (no vs. yes)	NA	NA	1.610 (1.051–2.466)	0.029
Tumor size, cm (≤5 vs. >5)	1.554 (1.103–2.189)	0.012	1.293 (0.964–1.736)	0.087
Microvascular invasion (no vs. yes)	1.241 (0.873–1.766)	0.229	1.391 (1.030–1.880)	0.031
Tumor differentiation (I-II vs. III-IV)	1.278 (0.865–1.887)	0.219	1.241 (0.887–1.737)	0.208
TNM stage (I vs. II III)	1.823 (1.242–2.677)	0.002	1.628 (1.159–2.289)	0.005
PKM2 (low vs. high)	1.572 (1.053–2.348)	0.027	1.593 (1.115–2.276)	0.010

Abbreviations: TTR, time to recurrence; OS, overall survival; AFP, α-fetoprotein; γ-GT, γ-glutamyl transferase; TNM, tumor-nodes-metastasis; HR, hazard ratio; CI, confidential interval; NA, not adopted; NS, not significant;
†Cox proportional hazards regression.

Supplementary Table S4: Sequence of primers for qRT-PCR

Gene	Forward primer (5′–3′)	Reverse primer (5′–3′)
PKM2	ATTATTTGAGGAACTCCGCCGCCT	ATTCCGGGTCACAGCAATGATGG
β-actin	CAACTGGGACGACATGGAGAAAAT	CCAGAGGCGTACAGGGATAGCAC
MIF	CTCTCCGAGCTCACCCAGCAG	CGCGTTCATGTCGTAATAGTT
S100A8	ATGCCGTCTACAGGGATGAC	ACTGAGGACACTCGGTCTCTA
CXCL1	ATGGCCCGCGCTGCTCTCTCC	CTTAACTATGGGGGATGCAGG
CCL2	CAGCCAGATGCAATCAATGCC	TGGAATCCTGAACCCACTTCT
CCR2	TGCAAAAAGCTGAAGTGCTTG	CAGCAGAGTGAGCCCACAAT
CCL3	AGTTCTCTGCATCACTTGCTG	CGGCTTCGCTTGGTTAGGAA
CCL4	ACCCTCCCACCGCCTGCTGCTTTT CTT AC	GTTCCAGGTCATACACGTACTCCT GGACCC
CCL5	CCAGCAGTCGTCTTTGTCAC	CTCTGGGTTGGCACACACTT
CCL8	TGGAGAGCTACACAAGAATCACC	TGGTCCAGATGCTTCATGGAA
CCL19	CTGCTGGTTCTCTGGACTTCC	AGGGATGGGTTTCTGGGTCA
CCL21	GTTGCCTCAAGTACAGCCAAA	AGAACAGGATAGCTGGGATGG
CCL22	ATCGCCTACAGACTGCACTC	GACGGTAACGGACGTAATCAC

Supplementary Table S5: Primary antibodies for western blot, IHC and co-IP

Antibody	Concentration for WB	Concentration for IHC	Concentration for IF	Concentration for FACS	Specificity	Company
PKM2	1:1000	1:100			Rabbit polyclonal	Cell Signaling Technology
PKM2			1:100		Rabbit polyclonal	Proteintech
PKM1/M2	1:1000				Rabbit monoclonal	Cell Signaling Technology
CD45				1:100	Rat monoclonal	Ebioscience
Gr-1		1:100	1:100	1:100	Rat monoclonal	Ebioscience
CD11b				1:100	Rat monoclonal	Ebioscience
F4/80		1:100		1:100	Rat monoclonal	Ebioscience
L6yC				1:100	Rat monoclonal	BD Biosciences
α-SMA		1:100	1:100		Mouse monoclonal	Dako
HIF-1α	1:2000				Rabbit monoclonal	Abcam
p-AKT	1:1000				Rabbit polyclonal	Cell Signaling Technology
AKT	1:1000				Rabbit polyclonal	Cell Signaling Technology
p-JNK	1:1000				Rabbit polyclonal	Cell Signaling Technology
JNK	1:1000				Rabbit polyclonal	Cell Signaling Technology
p-FAK	1:1000				Rabbit polyclonal	Cell Signaling Technology
FAK	1:1000				Rabbit polyclonal	Cell Signaling Technology
p-STAT3	1:1000				Rabbit polyclonal	Cell Signaling Technology
STAT3	1:1000				Rabbit polyclonal	Cell Signaling Technology
p-ERK1/2	1:1000				Rabbit polyclonal	Cell Signaling Technology
ERK	1:1000				Rabbit polyclonal	Cell Signaling Technology
β-ACTIN	1:5000				Mouse monoclonal	Sigma

Abbreviations: WB, western blot; IHC, immunohistochemistry; FACS, fluorescence activated cell sorter; IF, immunofluorescence.
